# Feasibility and Diagnostic Utility of Antigen-Specific Interferon-γ Responses for Rapid Immunodiagnosis of Tuberculosis Using Induced Sputum

**DOI:** 10.1371/journal.pone.0010389

**Published:** 2010-04-28

**Authors:** Tamaryn J. Cashmore, Jonathan G. Peter, Richard N. van Zyl-Smit, Patricia L. Semple, Alice Maredza, Richard Meldau, Alimuddin Zumla, Barbara Nurse, Keertan Dheda

**Affiliations:** 1 Lung Infection and Immunity Unit, Division of Pulmonology, UCT Lung Institute and Clinical Immunology, Department of Medicine, University of Cape Town, Cape Town, South Africa; 2 Department of Clinical Laboratory Sciences, National Health Laboratory Service/University of Cape Town, Cape Town, South Africa; 3 Institute of Infectious Diseases and Molecular Medicine, University of Cape Town, Cape Town, South Africa; 4 Centre for Infectious Diseases and International Health, Department of Infection, UCL Medical School, London, United Kingdom; MRC Laboratories, Gambia

## Abstract

**Background:**

The diagnosis of smear-negative or sputum-scarce tuberculosis (TB) is problematic as culture takes several weeks and representative biological samples are difficult to obtain. RD-1 antigen-specific interferon-γ release assays (IGRAs) are sensitive and specific blood-based tests for the diagnosis of *M. tuberculosis* infection. The feasibility and diagnostic utility of this rapid immunodiagnostic assay, using cells from induced sputum, is unknown.

**Methodology/Principal Findings:**

Cells isolated from induced sputum were co-cultured with ESAT-6 and CFP-10 antigens using a standardized enzyme-linked immunospot (ELISPOT) assay (T-SPOT®.*TB*) in 101 consecutively recruited TB suspects or non-TB controls. An optimization phase using 28 samples was followed by a validation phase using samples from 73 participants (20 with definite or probable TB, and 48 with non-TB). Despite optimization of sputum processing 65/73 (89%) of the IGRAs in the validation phase were inconclusive. 44/73 (60%) tests failed due to sputum induction-related factors [sputum induction-related adverse events (n = 5), inadequate sputum volume (n = 8), non-homogenisable sputum (n = 7), and insufficient numbers of cells to perform the assay (n = 24)], whilst 20/73 (27%) tests failed due T-SPOT®.*TB* assay-related factors [excessive debris precluding reading of spots in the ELISPOT well (n = 6), failure of the positive control (n = 11), or high spot count in the negative control (n = 3)]. Only 8/73 (11%) of the available samples could therefore be correctly categorized (7 definite or probable TB, and 1 non-TB patient). Thus, 13/20 (65%) of the definite or probable TB cases remained undiagnosed.

**Conclusions/Significance:**

Rapid immunodiagnosis of pulmonary TB by antigen-specific IFN-γ ELISPOT responses, using cells from induced sputum, is possible. However, the test, in its current ELISPOT format, is not clinically useful because the majority of the assays are inconclusive.

## Introduction

Tuberculosis (TB) has become a public health catastrophe in the developing world. The most recent WHO figures estimate a worldwide TB incidence of 9.27 million per year and 1.8 million deaths annually [1]. TB control efforts are hampered by suboptimal diagnostic tools and techniques. The sensitivity of routine smear-microscopy is approximately 50% [2], culture techniques take several weeks to yield results, and suitable representative biological samples are frequently unobtainable either due to lack of sputum production or poor sample quality. The HIV pandemic compounds this problem by increasing the incidence of smear-negative and sputum-scare TB [3].

Alternative techniques to obtain pulmonary samples are required in these patients including sputum induction (SI), gastric washings and bronchoscopy [4], [5]. However, bronchoscopy is expensive, invasive and not widely available in resource-limited settings, while gastric washing is largely limited to use in children. In contrast, sputum induction is non-invasive, less costly, has fewer side effects and has been shown to provide an equal, or higher microbiological yield when compared to bronchoscopy [6], [7], [8]. In resource-limited settings, therefore, the use of sputum induction could provide an ideal patient-friendly option. Nevertheless, rapid diagnosis of *Mycobacterium tuberculosis (M.tb)* is still frequently impossible given the low yield of smear microscopy, approximately 7–32% in this context [9], [10].

More recently the interferon-γ release assays (IGRAs) based on peripheral blood mononuclear responses to *M.tb* region of difference-1 (RD-1)-specific antigens [early secretory antigenic target-6 (ESAT-6) and culture filtrate protein-10 (CFP-10)], have been shown to be highly sensitive and specific tests for the diagnosis of *M.tb* infection [11], [12]. In addition, the standardised RD-1 ELISPOT assay (T-SPOT®.*TB*) has also shown to be an accurate tool for the rapid diagnosis of active TB using cerebrospinal fluid mononuclear cells [13], and broncho-alveolar lavage cells [14], [15]. Thus, the ELISPOT assay can be accurately used to diagnose active TB using cells from the site of disease.

We hypothesized that performing an IGRA, using mononuclear cells from induced sputum, could enable rapid immunodiagnosis of TB in a more patient-friendly and cost-effective manner. The feasibility and diagnostic utility of this approach using RD-1 antigen-specific ELISPOT responses has hitherto not been evaluated.

## Materials and Methods

### Clinical recruitment

**Figure 1:**
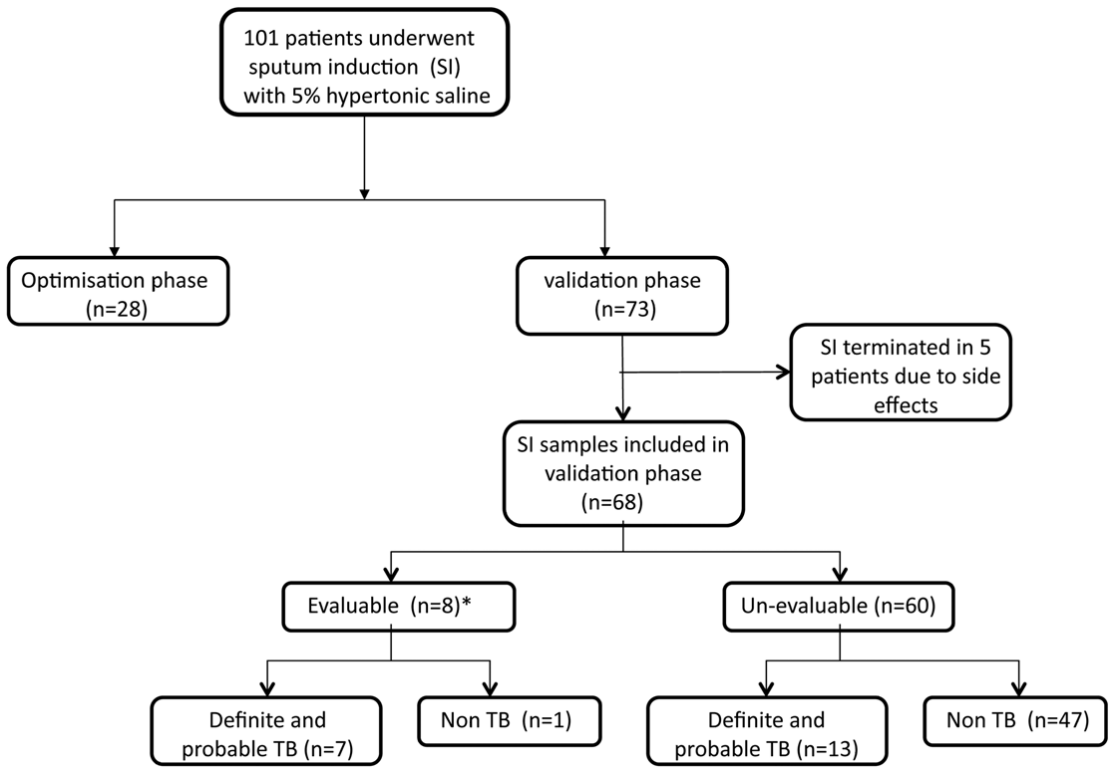
Study flow diagram. Study plan and patient categorisation of the 28 and 73 participants evaluated in the optimization and validation phases, respectively.

Eighty-one adults with suspected TB and twenty non-TB controls (8 with COPD or asthma, 6 with interstitial lung disease, 2 with cystic fibrosis, 2 with bronchiectasis, and 2 with lung cancer) were recruited from primary health care clinics in suburban Cape Town, and the Respiratory Clinic at Groote Schuur Hospital, South Africa between March 2008 and March 2010. (Figure 1) All patients had a chest x-ray and sputum induction performed. Standard short course anti-TB therapy was administered, when appropriate, according to local guidelines.

Ethical approval was obtained from the University of Cape Town's Health Science Faculty Research Ethics Committee (REC number 421/2006). Each patient gave written informed consent prior to participation in the study.

### Sample acquisition and processing

#### Sputum induction

All participants were requested to rinse their mouths with water and then be seated in an enclosed negative-pressure sputum induction booth. Approximately 20 ml of sterile 5% hypertonic saline (Sabax, Adcock Ingram Critical Care (PTY) LTD, Johannesburg, South Africa) was delivered via a Wilson's 402A ultrasonic nebuliser (Medimark, South Africa) over a period of 15 minutes or until 4–12 ml of induced sputum could be collected in a sterile container. No pro-expectorating maneuvers, such as chest percussion, were employed. Induction was immediately terminated if side effects such as dyspnoea, chest discomfort or nausea were reported. Strict infection control measures were followed during sample acquisition and processing. To avoid confounding, the same technician performed all laboratory assays and was blinded to the patient's final diagnosis.

#### Optimisation of the ELISPOT assay using cells from induced sputum

Given the lack of established processing methods, using induced sputum cells in an ELISPOT assay, an optimization phase using 28 patient samples was undertaken (see figure 1). To determine the optimal method for sputum homogenization different volumes of 0.1% dithiothreitrol (DTT) (Sigma, UK) were added to sputum samples, and the volume and rolling time determined by the sample's final macroscopic appearance, viability of the induced sputum mononuclear cells (trypan blue method), and the degree of background discoloration in the ELISPOT well. In summary, a 2∶1 volume ratio of 0.1% DTT to induced sputum with a rolling time of 20 minutes was sufficient to digest all mucins with minimal cell death and background well discoloration of the assays.

After homogenization and cell counting (Neubauer Haemacytometer) the sample was filtered either through 2-ply sterile gauze [22% (range 0–54%) loss of mononuclear cells], a 100 µm cell strainer [56% (range 17–100%) cell loss]or using a ficoll density gradient centrifugation [93% (range 85–99%) cell loss]. Thus, using sterile gauze was determined to be the optimal filtration method.

**Figure 2:**
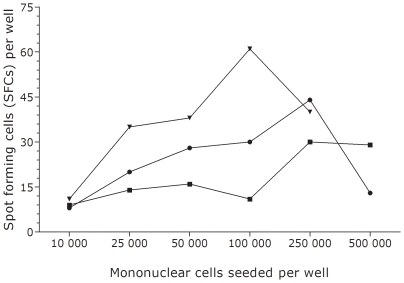
Optimization of mononuclear sputum cell number. Trend lines represent mean numbers of spots in ESAT-6-stimulated wells using cells from smear-positive individuals. All positive and negative controls were valid.

To determine the optimal number of cells to be used per ELISPOT well, mononuclear induced sputum cells from 3 smear-positive subjects with TB were plated in duplicate wells at several concentrations. 250 000 cells/well was determined to be the optimal cell number to be used per well with a further increase in cell number per well diminishing antigen-specific responses (Figure 2).

#### Sample processing for validation phase

Seventy-three patients were included in the validation phase (Figure 1). Samples were processed according to methods derived from the optimization phase (dilution with 0.1% DTT twice the sputum volume, homogenization and rolling for 20 minutes at room temperature, and filtration through 2-ply sterile gauze). A 2–6 ml aliquot was removed and sent for microbiological testing. The sample was then washed twice with BioWhittaker® 10% phosphate buffered saline (Lonza, Walkersville, USA) and cells isolated by centrifugation at 300×g for 10 minutes. Mononuclear cells were resuspended in 1–2 ml AIM-V® medium (Gibco, Invitrogen) counted, viability assessed and plated in duplicate in the ELISPOT assay.

### Enumeration of RD-1 specific T cells

The T-SPOT®.*T*B assay was performed as per the manufacturer's instructions. Spot forming units/10^6^ cells (SFUs), representing IFN-γ release by peptide-specific T cells, were counted with an automated AID-ELISPOT reader system (Autoimmun Diagnostika, Strassberg, Germany), and confirmed by manual counting. The diagnosis of TB was based on the number of SFUs according to the manufacturer's guidelines for peripheral blood mononuclear cells.

### Diagnostic classification

Smear-microscopy was performed on centrifuged and processed sputum using fluorescence microscopy, and sputum quality was assessed using Gram staining. The following criteria were used for final diagnostic classification:

- Definite TB – positive culture for *M. tuberculosis* (MGIT 960 platform).
- Probable TB – TB highly likely and the patient treated for TB on clinical or radiological grounds, but in the absence of a confirmed microbiological diagnosis.
- Non-TB – smear microscopy and culture negative, no chest x-ray evidence of active TB, and not treated for tuberculosis, or successful treatment for an alternative infection. The subject remained healthy at follow-up (6 months).

### Data analyses

Statistical analysis was performed using STATA version 8. GraphPad Prism (version 4.0 or higher) was used for graphs and figures.

## Results

### Patient demographics & clinical characteristics

The demographic characteristics of the seventy three subjects included in the validation phase are shown in table 1. The HIV prevalence was 29%. Sixteen (23%) patients had definite TB, 4 (6%) probable TB and 48 (71%) non-TB. There were no significant differences between the definite and non-TB groups.

**Table 1:** Demographic information of patients included in the validation phase excluding 5 patients who were un-inducible.

	Total	Definite TB	Probable TB	Non-TB
**Number of subjects**	68	16(23%)	4 (6%)	48 (71%)
**Age** mean(SD)	46 (17)	38 (15)	48 (10)	49 (17)
**Sex**				
Male	33(49%)	10(63%)	3(75%)	20(42%)
**Racial group**				
Black African	29(43%)	9 (56%)	3(75%)	17(35%)
Mixed Ancestry	35(51%)	7 (44%)	1(25%)	27(56%)
European Ancestry	4(6%)	0	0	4(9%)
**Current smoker**	20 (29%)	5 (31%)	2(50%)	13(27%)
**HIV status**				
positive	20 (29%)	7 (44%)	3(75%)	10 (21%)
unknown/refused testing	10 (15%)	0	0	10 (21%)
**Previous TB (n = 54)**	16(24%)	5(31%)	2(50%)	9(19%)

### Sputum induction and processing

The median duration of sputum induction was 15 minutes (range: 5–20 minutes). Periods of less than 15 minutes were deemed acceptable if a suitable sample was obtained. Five (6%) of the total 73 sputum inductions were prematurely terminated (nausea and/or vomiting in 2 patients and chest pain in 3 patients). The median volume of induced sputum was 7 ml (range: 4–12 ml) while 8 patients produced ≤1 ml which was considered inadequate for further processing (a summary of test outcomes and reasons for un-evaluable results is presented in Table 2). Due to the presence of large mucus plugs 7 samples could not be successfully homogenised, and thus could not be adequately filtered. Amongst samples successfully homogenised, filtered and washed the median (range) sputum cell concentration (cells/milliliter sputum) was 0.1×106 cells/ml (0–2.4×106), and the viability greater than 90%. The differential cell counts showed a median (range) lymphocyte count of 21% (0–75%) and neutrophil count of 79% (10–95%). Scanty macrophages were occasionally detected. Female patients produced significantly less sputum volumes and consequently sputum cell concentrations (p = 0.027 and p = 0.038, respectively). There was no correlation between induced sputum volumes, cell concentrations or age, and HIV status. Total sputum cell numbers were highly variable and in 23 patients there was insufficient cells to perform the T-SPOT®.*TB* assay (Table 2) while in a further 19 cases the samples' total cell yields were insufficient to plate the optimal 250 000 cells/well, and consequently these assays we performed using concentrations above 100 000 cells/well.

**Table 2:** T-SPOT®.*TB* assay outcomes and reasons for inconclusive test results in the validation phase.

Validation phase (n = 68)+ Test outcomes/reasons for test failures	Definite and Probable TB (n = 20)+	Non TB *(n = 48)
Sputum induction-related factors (n = 39)		
1. Inadequate volume of sputum	1	7
2. Failure to homogenise sputum	1	6
3. Insufficient cells	6	18
T-SPOT®.*TB* related factors (n = 20)		
1. Excessive debris (high background)	1	5
2. Positive control failed	1	10
3. Negative control failed	2	1
Valid T-SPOT®.*TB* result (n = 8)		
1. Positive	7#	0
2. Negative	0	1

*The ‘non-TB’ group included i) 29 TB suspects classified as non TB after follow-up and ii) non TB controls patients with alternative respiratory diseases (e.g. interstitial lung disease).

# 6 positive T-SPOT®.TB from definite *TB* patients and 1 positive T-SPOT®.*TB* from probable TB patient.

+ 1 sample rejected due to laboratory labeling error.

### ELISPOT assay

**Figure 3:**
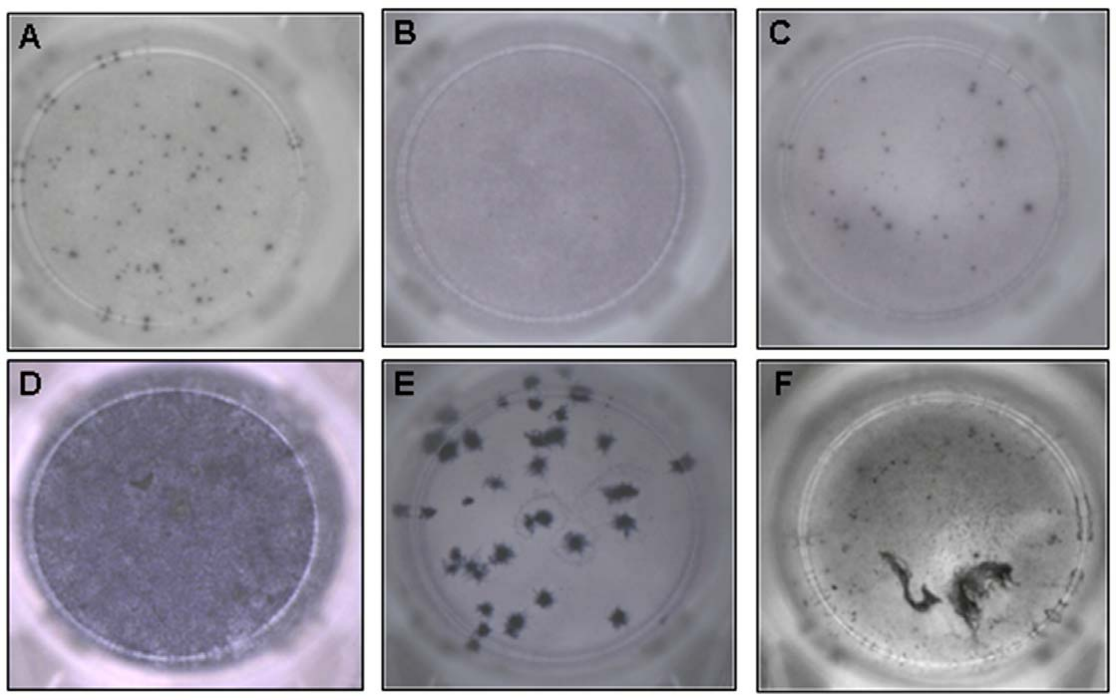
AID® ELISPOT reader images of T-SPOT®.*TB* wells demonstrating. (A) positive control, (B) valid negative control, (C) positive ESAT-6 well, (D) high background discoloration and (E,F) artefacts from non-specific debris and mucus.

Of the 73 patients included in the validation phase, after processing only 29 samples had a sufficient total cell number to warrant performing the T-SPOT®.*TB* assay (Table 2). Only eight of these 29 samples produced evaluable T-SPOT®.*TB* results. The main reasons for unevaluable T-SPOT®.*TB* assay results are outlined in table 2 and photographic examples are shown in Figure 3A–F.

Thus, overall only 8/73 (11%) of the samples collected yielded an evaluable T-SPOT®.*TB* result. Of these 7 were positive with a median(IQR) of 96(21,164) SFUs for the ESAT-6 and 64 (0,250) CFP-10 wells, respectively. All 7 positive samples were from TB patients and the one negative sample was from a non TB patient. The relationship between evaluable T-SPOT®.*TB* results and AFB smear and culture results are illustrated in table 3.

**Table 3:** Characteristics of the 8/73 (11%) interpretable T-SPOT®.*TB* assays.

		Spot forming units/10^6^cells				SI smear/culture	
T-SPOT®.*TB* result	Final diagnosis	ESAT-6	CFP-10	Positive control*	Negative control*	AFB smear	MGIT culture
positive	Definite TB	120	not done#	100	40	positive	positive
positive	Definite TB	176	not done#	64	40	positive	positive
positive	Definite TB	160	not done#	80	20	positive	positive
positive	Definite TB	28	>250	>250	0	negative	positive
positive	Definite TB+	0	>250	>250	0	negative	negative
positive	Definite TB+	>250	0	60	0	negative	negative
positive	Probable TB	72	64	>250	12	negative	negative
negative	Non TB	0	0	40	0	not done$	not done$
Median		96	64	90	0		
IQR		21/164	0/250	63/250	0/25		

*As per the manufacturer's guidelines, an assay was considered valid if the number of SFU's/106cells in the negative control well was twice that of the positive control well. A value of >250SFU's/106cells was selected as the cut-off positive value. ESAT-6  =  Early Secretory Antigen Target-6. CFP-10  =  Culture Filtrate Protein-10. SI  =  Induced sputum sample.

# Insufficient cells to plate both wells.

+ Patients were found to be culture positive on other biological samples and therefore classified as definite TB.

$ Asymptomatic control patient with COPD and normal chest x-ray (sputum smear and culture was not indicate.

## Discussion

We evaluated the diagnostic utility of induced sputum mononuclear cells in the RD-1 ELISPOT assay as a rapid immunodiagnostic test for active tuberculosis. However, only 8/73 (11%) collected samples yielded evaluable test results. Nevertheless, despite the major technical difficulties and frequent invalid results, the assay was highly sensitive in the limited number of evaluable results. Our study, using a standardized ELISPOT assay incorporating RD-1 specific antigens (ESAT-6 and CFP-10), confirms that rapid immunodiagnosis of *M.tb* infection is indeed possible using mononuclear cells from induced sputum, but demonstrates that due to technical factors its clinical utility is limited.

In contrast to our findings Breen *et al*. demonstrated, in a limited number of patients, that induced sputum could successfully be used for immunological diagnosis of TB using purified protein derivative (PPD)-driven cells in a flow cytometric or ELISPOT assay [7]. However, the ELISPOT assay which can be interpreted using a magnifying lens and with minimal training, obviates the need for technical competence in flow cytometry and costly instrumentation such as a flow cytometer, making it more appealing for use in a developing world setting. Moreover, they studied PPD-associated responses which are non-specific and detectable even in patients with LTBI. By contrast RD-1 specific responses in the lung are highly specific even in high burden countries where the prevalence of LTBI is over 50% [14], [16]. Breen *et al*. produced evaluable test results in all 9 study patients in whom ELISPOT was performed and total cell yield from induced sputum samples were almost 6 times greater than those found in our study. How do we explain these results? We used unselected and consecutively recruited patients, a different population group, and different sputum processing methodology. Several other factors including disease extent, the small number of patients evaluated by ELISPOT in the Breen study, the duration of sputum induction and volume of hypertonic saline used, and malnutrition and hence attenuated immune responses, may explain the discordant results.

The key drawback of the ELISPOT assay when using cells from induced sputum was the high proportion (89%) of inconclusive results. Insufficient cell yield (31%) was the most common reason for non-evaluable assays. This is not surprising as sputum induction samples the large airways rather than the alveolar spaces where the majority of the mononuclear cells reside. The low cellular yield is exacerbated by the variable though low proportion of lymphocytes in pulmonary samples. Increasing the numbers of cells per well is unlikely to have helped as demonstrated by the cell-cytokine response curves (Figure 2). We ensured maximal sample yield through using at least 20 ml of hypertonic saline and nebulising for at least 15 minutes. The volume and number of mononuclear cells isolated from the induced sputum samples in our study was less in females, an association previously reported [17] but did not correlate with age or HIV positivity. Therefore, neither obtaining a larger sample volume, nor exposing the patient to sputum induction for longer, is likely to overcome the problem of insufficient cells. Furthermore, cell viability was greater than 90% in all samples processed and consequently this factor is unlikely to have contributed to the poor test outcomes.

Other factors accounting for the inconclusive results included high IFN-γ readouts in the negative control well and high background discoloration of the wells likely related to the high protein and mucus content of induced sputum. It may be argued that one or more of these factors could be reduced thereby improving the diagnostic utility and feasibility. However, we believe this is unlikely for several reasons. Increasing the length of mixing time after addition of DTT failed to improve sample quality. Moreover, previous studies [8], [18], [19] suggest a high degree of spontaneous IFN-γ release by mononuclear cells, in the absence of any exogenous stimuli, especially those compartmentalized to the lungs of patients. Finally, several steps were taken to minimise cellular and other debris.

A limitation of our study was that concurrent blood and induced sputum assays were not performed, however, the limited utility and poor specificity of peripheral blood responses for active TB have been clearly demonstrated in high burden settings [12]. It is possible, though unlikely, that population-specific factors may have played a role and thus our findings require confirmation in different geographical settings.

In summary, we have confirmed that antigen-specific ELISPOT responses can be detected using cells from induced sputum for the diagnosis of TB. However, due to several technical factors the proportion of inconclusive results is too high to warrant current clinically utility. Further studies are now required to improve the assay formats and technologies used so that in the future rapid immunodiagnosis using cells from induced sputum may be feasible.

## References

[1] World Health Organisation (2009).

[2] Steingart KR, Ramsay A, Pai M (2007). Optimizing sputum smear microscopy for the diagnosis of pulmonary tuberculosis.. Expert Rev Anti Infect Ther.

[3] Moore DA, Roper MH (2007). Diagnosis of smear-negative tuberculosis in people with HIV/AIDS.. Lancet.

[4] Schoch OD, Rieder P, Tueller C, Altpeter E, Zellweger JP (2007). Diagnostic yield of sputum, induced sputum, and bronchoscopy after radiologic tuberculosis screening.. Am J Respir Crit Care Med.

[5] Brown M, Varia H, Bassett P, Davidson RN, Wall R (2007). Prospective study of sputum induction, gastric washing, and bronchoalveolar lavage for the diagnosis of pulmonary tuberculosis in patients who are unable to expectorate.. Clin Infect Dis.

[6] Conde MB, Soares SL, Mello FC, Rezende VM, Almeida LL (2000). Comparison of sputum induction with fiberoptic bronchoscopy in the diagnosis of tuberculosis: experience at an acquired immune deficiency syndrome reference center in Rio de Janeiro, Brazil.. Am J Respir Crit Care Med.

[7] Breen RA, Hardy GA, Perrin FM, Lear S, Kinloch S (2007). Rapid diagnosis of smear-negative tuberculosis using immunology and microbiology with induced sputum in HIV-infected and uninfected individuals.. PLoS ONE.

[8] Robinson BW, McLemore TL, Crystal RG (1985). Gamma interferon is spontaneously released by alveolar macrophages and lung T lymphocytes in patients with pulmonary sarcoidosis.. J Clin Invest.

[9] McWilliams T, Wells AU, Harrison AC, Lindstrom S, Cameron RJ (2002). Induced sputum and bronchoscopy in the diagnosis of pulmonary tuberculosis.. Thorax.

[10] Morse M, Kessler J, Albrecht S, Kim R, Thakur R (2008). Induced sputum improves the diagnosis of pulmonary tuberculosis in hospitalized patients in Gaborone, Botswana.. Int J Tuberc Lung Dis.

[11] Dheda K, Udwadia ZF, Huggett JF, Johnson MA, Rook GA (2005). Utility of the antigen-specific interferon-gamma assay for the management of tuberculosis.. Curr Opin Pulm Med.

[12] Pai M, Zwerling A, Menzies D (2008). Systematic review: T-cell-based assays for the diagnosis of latent tuberculosis infection: an update.. Ann Intern Med.

[13] Patel V, Singh R, Connelly C, Coovadia Y, Peer A (2010). Cerebrospinal T-cell responses aid the diagnosis of tuberculous meningitis in a HIV and TB endemic population.. AJRCCM (in press).

[14] Jafari C, Thijsen S, Sotgiu G, Goletti D, Dominguez Benitez JA (2009). Bronchoalveolar Lavage Enzyme-linked Immunospot for a Rapid Diagnosis of Tuberculosis: A TBNET Study..

[15] van Zyl-Smit R, Meldau R, Dheda K Quantitative pulmonary T-cell responses for the diagnosis of active tuberculosis.. Am J Respir Crit Care Med.

[16] Dheda K, van Zyl-Smit RN, Meldau R, Meldau S, Symons G (2009). Quantitative lung T cell responses aid the rapid diagnosis of pulmonary tuberculosis.. Thorax.

[17] Gonzalez AV, Menzies D (2008). In women with suspected TB, brief sputum-submission instruction improved sampling quality and TB detection.. Evid Based Med.

[18] Martinez-Maza O, Andersson U, Andersson J, Britton S, De Ley M (1984). Spontaneous production of interferon-gamma in adult and newborn humans.. J Immunol.

[19] Quiding M, Granstrom G, Nordstrom I, Ferrua B, Holmgren J (1993). High frequency of spontaneous interferon-gamma-producing cells in human tonsils: role of local accessory cells and soluble factors.. Clin Exp Immunol.

